# New online dynamic nomograms to predict recurrence-free and overall survival after resection of endometrial cancer: a single-institution retrospective cohort study

**DOI:** 10.1007/s00404-024-07596-x

**Published:** 2024-06-17

**Authors:** Zhen Hu, Junying Li, Junqiang Du

**Affiliations:** Gynecology and Obstetrics Unit, Dongyang Hospital of Wenzhou Medical University, 60 Wuning West RoadDongyang City, Jinhua City, Zhejiang Province China

**Keywords:** FIGO (Federation of Gynecology and Obstetrics) staging, Lymphatic vessel space invasion, Prognosis, Dynamic nomogram, Endometrial cancer

## Abstract

**Purpose:**

The significant global burden of endometrial cancer (EC) and the challenges associated with predicting EC recurrence indicate the need for a dynamic prediction model. This study aimed to propose nomograms based on clinicopathological variables to predict recurrence-free survival (RFS) and overall survival (OS) after surgical resection for EC.

**Methods:**

This single-institution retrospective cohort study included patients who underwent surgical resection for EC. Web-based nomograms were developed to predict RFS and OS following resection for EC, and their discriminative and calibration abilities were assessed.

**Results:**

This study included 289 patients (median age, 56 years). At a median follow-up of 51.1 (range, 4.1–128.3) months, 13.5% (39/289) of patients showed relapse or died, and 10.7% (31/289) had non-endometrioid tumors (median size: 2.8 cm). Positive peritoneal cytology result (hazard ratio [HR], 35.06; 95% confidence interval [CI], 1.12–1095.64; *P* = 0.0428), age-adjusted Charlson comorbidity index (AACCI) (HR, 52.08; 95% CI, 12.35–219.61; *P* < 0.001), and FIGO (Federation of Gynecology and Obstetrics) stage IV (HR, 138.33; 95% CI, 17.38–1101.05; *P* < 0.001) were predictors of RFS. Similarly, depth of myometrial invasion ≥ 1/2 (HR, 1; 95% CI, 0.46–2.19; *P* = 0.995), AACCI (HR, 93.63; 95% CI, 14.87–589.44; *P* < 0.001), and FIGO stage IV (HR, 608.26; 95% CI, 73.41–5039.66; *P* < 0.001) were predictors of OS. The nomograms showed good predictive capability, positive discriminative ability, and calibration (RFS: 0.895 and OS: 0.891).

**Conclusion:**

The nomograms performed well in internal validation when patients were stratified into prognostic groups, offering a personalized approach for risk stratification and treatment decision-making.

## What does this study add to the clinical work

**Table:** 

This study introduces novel online nomograms based on three variables to dynamically predict recurrence-free survival (RFS) and overall survival (OS) in women with endometrial cancer (EC). These nomograms offer a personalized approach for clinicians to stratify patients into different predictive groups based on their risk of relapse and long-term outcomes, aiding in the design of tailored clinical treatments. Furthermore, the nomograms demonstrated superior performance and discriminative power, providing vital prognostic information for gynecologists managing EC patients.	

## Introduction

Endometrial cancer (EC) is the sixth most frequently reported cancer in women worldwide, accounting for an estimated 417,000 new cases and 97,000 fatalities among women in 2020. The incidence of EC varies significantly, with tenfold difference observed between countries with high human development index (HDI) and those with low HDI. It is projected that by 2040, there will be a 47% increase in death rates globally due to population growth and aging, potentially exacerbating the situation [1]. Despite this, most patients with EC survive the initial diagnosis, making them the largest group of survivors among those with gynecological malignancies. However, relapse remains the primary cause of death in these patients [2]. Currently, most models of predicting EC recurrence rely on conventional clinicopathological variables [3]. The nomogram model developed by Ouldamer can predict poor prognosis based on histological type and grade, age, lymphatic vessel space invasion (LVSI), FIGO (Federation of Gynecology and Obstetrics) stage, and surgical nodal staging in patients with stages I–III EC [2]. Additionally, Takahashi et al. [4] recently developed a scoring system for predicting recurrence in stages I–II EC based on various factors, such as age, pathology, cervical stromal invasion, and peritoneal cytology. Nevertheless, additional predictive indicators are warranted to optimize such models [5].

A reliable prognostic marker is essential for guiding physicians in determining the requisite adjuvant therapy and follow-up for patients after cancer resection. While FIGO or NCCN classification may be helpful for general survival predictions [6, 7], individual patient prognoses may benefit from risk stratification systems. With the advent of individualized tumor treatment, nomograms can be advantageous and serve as a safe and comprehensible statistical tool that can combine multiple factors to help physicians make individualized patient assessment, treatment decisions, and follow-up choices. To the best of our knowledge, no such dynamic prediction model is available for EC. Therefore, this study aimed to develop an online dynamic nomogram to help physicians provide personalized treatment and follow-up guidance.

## Methods

### Data collection and patient population

In this single-institution retrospective cohort study, we identified patients who underwent surgical resection for EC between January 1, 2012, and 31 August 2022 at Dongyang People’s Hospital of Wenzhou Medical University, Zhejiang, China. The follow-up termination date was December 31, 2022. Data were collected using the medical record mining software of Shanghai Le9 Healthcare Technology Co., Ltd (Le9 Healthcare Technology, Shanghai, China). This study only included patients who underwent surgery as a treatment modality. Patients aged < 18 years and those with missing data were excluded. Additionally, patients who did not survive beyond 1 month postoperatively were excluded to prevent the incorporation of postoperative complications. Based on the inclusion and exclusion criteria, 5 patients with missing surgical and pathological data, 0 patients aged < 18 years, and 0 patients who died within 1 month after surgery were excluded, and 289 patients were finally included in the cohort analysis. This study was approved by the Ethics Committee of Dongyang People’s Hospital of Wenzhou Medical University (approval number: DRY-2023-YX-078), and written informed consent was obtained from individual participants. The requirement of any additional consent was waived due to the retrospective nature of the study.

A survey of demographics and clinicopathological parameters was conducted, which included age at surgery, presence of adenomyosis, menopause, age-adjusted Charlson comorbidity index (AACCI), FIGO stage, histological type, primary tumor diameter, LVSI, histological grade, peritoneal cytology, depth of myometrial invasion, presence or absence of lymph node metastases, and cervical stromal invasion. Resection specimens were evaluated based on their maximum diameters, and tumors were categorized as either endometrioid or non-endometrioid. The myometrial invasion depth, the lymph node metastasis, and the cervical stromal invasion status were determined based on the final pathological assessment. LVSI was classified as negative, focal, or substantial. Outcome status was defined as cancer recurrence or cancer-related death. The primary outcomes were long-term overall survival (OS) and recurrence-free survival (RFS). High-risk patients received guideline-guided postoperative radiotherapy (mainly external beam radiotherapy and vaginal brachytherapy), chemotherapy (mainly paclitaxel plus carboplatin or cisplatin), or both. These treatment approaches may have partially influenced our estimates of RFS and OS.

## Statistical analysis

Continuous variables are presented as medians and interquartile ranges (IQRs) and categorical variables as complete values, unless specified otherwise. RFS and OS were determined using Kaplan–Meier curves, and comparisons were made using the log-rank test. Clinical and pathological factors associated with survival risk and recurrence were evaluated a priori [8–11]. Using a correlation matrix, were examined variable collinearity and interactions, including those between age, adenomyosis, menopause, AACCI, FIGO stage, histological type, primary tumor diameter, LVSI, histological grade, peritoneal cytology, depth of myometrial invasion, lymph node metastases, and cervical stromal invasion. Multivariate analysis did not include an interaction term due to lack of significant interaction. Linear correlation between continuous predictors (i.e., age and primary tumor diameter) and risk of recurrence or death was evaluated using restricted cubic splines to categorize the continuous predictors [12]. Age (56 years) and primary tumor diameter (2.8 cm) were associated with an increased risk of recurrence and death. Nomograms were constructed with tumor size and age as categorical variables for consistency according to prior research and clinical practice guidelines (< 2 vs. ≥ 2 cm and < 56 vs. ≥ 56 years, respectively) [6, 7, 11]. The association of clinicopathological factors with RFS and OS was identified using a Cox proportional risk regression model. Backward stepwise selection using the Akaike information criterion (AIC) was employed to develop multivariable Cox proportional hazards regression models. Hazard ratios (HRs) with 95% confidence intervals (CIs) were reported [13]. Using statistical software, specific factors were included in the nomograms to predict the 3-, 5-, and 10-year RFS and OS following EC surgical resection with curative intent (RMS in R, version 4.1.1; http://www.r-project.org). Linear predictors were defined by applying regression coefficients to each individual observation [14].

The performance of the model was evaluated based on its ability to accurately predict individual outcomes (discriminative ability) and estimate the survival function (calibration). Harrell et al. [15] evaluated the performance of nomogram using C-statistics, which is equivalent to the area under the curve (AUC) of the receiver operating characteristic (ROC) curve and can gage the agreement between the anticipated and actual results. The effectiveness of calibration was determined using a calibration plot, which represents the correlation between observed outcomes and predicted probabilities using a bootstrapped sample. Ideally, the line of prediction should align at a 45° angle. The clinical utility of the model was measured using decision curve analysis (DCA) [16]. Kaplan–Meier curves were drawn across the threshold for patients classified by nomogram scores in the dataset to assess the calibration further. Overfitting was quantified using bootstrapped resampling of the model, and improvement in model was judged using the integrated discrimination improvement (IDI) index [17]. Finally, a website was developed to enable clinicians to access and utilize the proposed paradigm. Two-tailed tests were employed, and statistical significance was defined as *P* < 0.05.

## Results

### Demographic and clinicopathological features

**Fig. 1:**
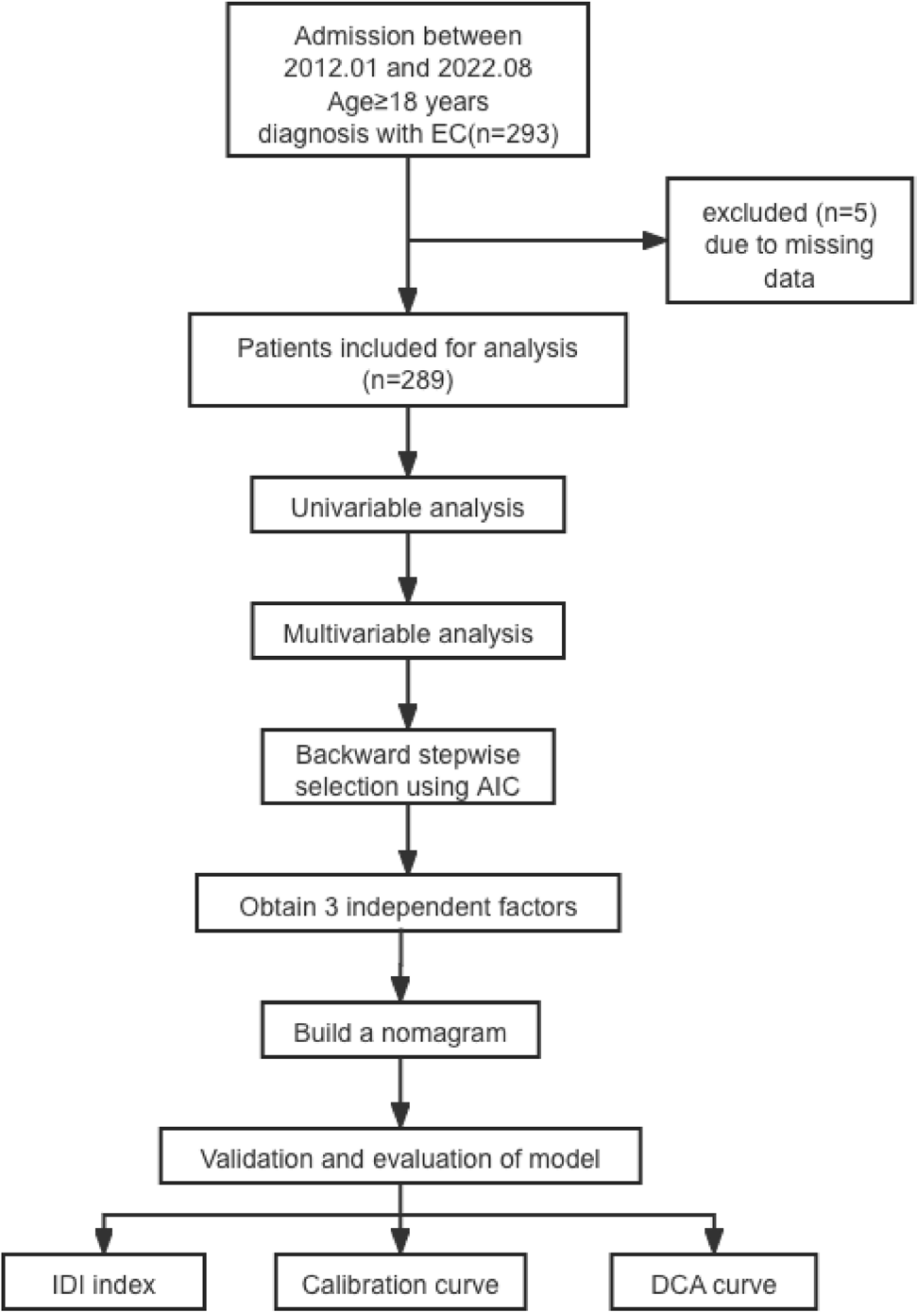
Flowchart of the study

The study flowchart is presented in Fig. 1. The median age of the participants was 56 years (IQR, 51–61 years), and the median AACCI was 1 (IQR, 1–3). Overall, 60.9% (176/289) of patients were in menopausal stage, and 13.8% (40/289) had adenomyosis. Further, only 0.7% (2/289) of the patients showed positive findings for peritoneal cytology, while 5.5% (16/289) showed positive finding for lymph node metastases. Regarding histological type, 10.7% (31/289) of the participants had non-endometrioid tumors. The overall pathology report indicated that the majority of the participants had early-stage tumors, with 83.4% (241/289) having stage I cancer, whereas 6.2% (18/289) having stage II cancer, 8.7% (25/289) having stage III cancer, and 1.7% (5/289) having stage IV cancer. Cervical stromal invasion was detected in 10.0% (29/289) of the patients. The median primary tumor diameter was 2.8 cm (IQR, 1.5–4.2 cm), and 19.0% (55/289) of the participants had deep myometrial invasion. High histological grade was observed in 16.6% (48/289) of the patients. Overall, 11.1% (32/289) of the patients showed LVSI-positive finding: 9.7% (28/289) had focal LVSI, whereas only 1.4% (4/289) had substantial LVSI (Online Resource 1).

At a median follow-up of 51.1 (range, 4.1–128.3) months, 13.5% (39 of 289) of the patients showed relapse or died from their disease. The unadjusted median RFS was 47.1 months (95% CI, 7.6–108.3), and the unadjusted median OS was 51.1 months (95% CI, 8.6–108.9). The 3- and 5-year RFS was 89.3% (95% CI, 85.7–93.3%) and 82.6% (95% CI, 77.5–88.0%), respectively, whereas the OS was 93.7% (95% CI, 90.7–96.8%) and 83.9% (95% CI, 78.7–89.5%), respectively (Online Resource 2).

## Model specifications and predictors of RFS and OS

Candidate variables for predictive models were selected based on tumor characteristics of clinical significance and demographics. Through AIC stepwise backward selection in the Cox proportional risk regression model, the following three variables exhibited the strongest correlation with OS: AACCI > 2, FIGO stage, and depth of myometrial invasion ≥ 1/2 (Table 1). Similarly, the following three variables were associated with recurrence risk: AACCI > 2, FIGO stage, and positive peritoneal cytology result (Table 2). Multivariable analysis revealed that AACCI > 2 (HR, 52.08; 95% CI, 12.35–219.61; *P* < 0.001), positive peritoneal cytology result (HR, 35.06; 95% CI, 1.12–1095.64; *P* = 0.0428), histological grade (HR, 2.95; 95% CI, 1.1–7.92; *P* = 0.0314), and FIGO stage IV (HR, 138.33; 95% CI, 17.38–1101.05; *P* < 0.001) were independently associated with RFS. Further, multivariable analysis revealed that AACCI > 2 (HR, 93.63; 95% CI, 14.87–589.44; *P* < 0.001), cervical stromal invasion (HR, 6.15; 95% CI, 1.02–36.96; *P* = 0.0472), primary tumor diameter ≥ 2 (HR, 6.28; 95% CI, 1.33–29.72; *P* = 0.0205), substantial LVSI (HR, 0.05; 95% CI, 0.01–0.38; *P* = 0.0043), and FIGO stage IV (HR, 608.26; 95% CI, 73.41–5039.66; *P* < 0.001) were independently associated with OS.

**Table 1:** Cox proportional hazards regression model showing the association of variables with overall survival

Variable	Univariable		Multivariable	
	HR (95% CI)	*P* value	HR (95% CI)	*P* value
Factors Selected				
AACCI				
≤ 2	1 [Reference]	NA	1 [Reference]	NA
> 2	23.07 (8.17–65.13)	< 0.001	93.63 (14.87–589.44)	< 0.001
FIGO				
I	1 [Reference]	NA	1 [Reference]	NA
II	2.79 (1.05–7.46)	0.04	0.21 (0.03–1.69)	0.1437
III	4.71 (2.07–10.71)	< 0.001	1.23 (0.35–4.36)	0.7441
IV	92 (28.97–292.2)	< 0.001	608.26 (73.41–5039.66)	< 0.001
Depth of myometrial invasion				
< 1/2	1 [Reference]	NA	1 [Reference]	NA
≥ 1/2	4.32 (2.28–8.17)	< 0.001	1 (0.46–2.19)	0.995
Factors Not Selected				
Histological grade				
1–2	1 [Reference]	NA	1 [Reference]	NA
3	4.98 (2.63–9.42)	< 0.001	2.19 (0.88–5.42)	0.0912
Primary tumor diameter, cm				
< 2	1 [Reference]	NA	1 [Reference]	
≥ 2	5.29 (1.63–17.22)	0.006	6.28 (1.33–29.72)	0.0205
Age at surgery, y				
< 56	1 [Reference]	NA	1 [Reference]	NA
≥ 56	2.42 (1.2–4.88)	0.014	0.42 (0.17–1.04)	0.0608
Cervical stromal invasion				
No	1 [Reference]	NA	1 [Reference]	NA
Yes	4.33 (2.18–8.59)	< 0.001	6.15 (1.02–36.96)	0.0472
Histological type				
Endometrioid	1 [Reference]	NA	1 [Reference]	NA
Non-endometrioid	4.52 (2.27–8.99)	< 0.001	1.08 (0.4–2.95)	0.8815
Lymph node metastases				
No	1 [Reference]	NA	1 [Reference]	NA
Yes	5.34 (2.33–12.25)	< 0.001	2.08 (0.52–8.32)	0.3021
LVSI				
Negative	1 [Reference]	NA	1 [Reference]	NA
Focal	5.13 (2.49–10.54)	< 0.001	2.25 (0.93–5.45)	0.0724
Substantial	17.42 (5.96–50.92)	< 0.001	0.05 (0.01–0.38)	0.0043
Peritoneal cytology				
Negative	1 [Reference]	NA	1 [Reference]	NA
Positive	21.79 (2.67–178.07)	0.004	1.68 (0.09–30.91)	0.7261
Menopause				
No	1 [Reference]	NA	1 [Reference]	NA
Yes	2.16 (0.99–4.71)	0.054	NA	NA
Adenomyosis				
No	1 [Reference]	NA	1 [Reference]	NA
Yes	0.19 (0.03–1.37)	0.099	NA	NA

AACCI: age-adjusted Charlson comorbidity index; FIGO: Federation of Gynecology and Obstetrics; HR: hazard ratio; LVSI: lymphatic vessel space invasion; NA, not applicable

**Table 2:** Cox proportional hazards regression model showing the association of variables with recurrence-free survival

Variable	Univariable		Multivariable	
	HR (95% CI)	*P* value	HR (95% CI)	*P* value
Factors Selected				
AACCI				
≤ 2	1 [Reference]	NA	1 [Reference]	NA
> 2	25.65 (9.1–72.3)	< 0.001	52.08 (12.35–219.61)	< 0.001
FIGO				
I	1 [Reference]	NA	1 [Reference]	NA
II	3.11 (1.17–8.25)	0.023	1.22 (0.18–8.25)	0.837
III	4.6 (2.03–10.42)	< 0.001	0.95 (0.23–3.96)	0.9434
IV	102.59 (29.66–354.82)	< 0.001	138.33 (17.38–1101.05)	< 0.001
Peritoneal cytology				
Negative	1 [Reference]	NA	1 [Reference]	NA
Positive	14.11 (1.87–106.36)	0.01	35.06 (1.12–1095.64)	0.0428
Factors Not Selected				
Age at surgery, y				
< 56	1 [Reference]	NA	1 [Reference]	NA
≥ 56	2.65 (1.32–5.33)	0.006	0.36 (0.15–0.88)	0.025
Histological grade				
1–2	1 [Reference]	NA	1 [Reference]	NA
3	5.8 (3.09–10.87)	< 0.001	2.95 (1.1–7.92)	0.0314
Cervical stromal invasion				
No	1 [Reference]	NA	1 [Reference]	NA
Yes	4.73 (2.39–9.35)	< 0.001	1.68 (0.35–8.11)	0.5194
Depth of myometrial invasion				
< 1/2	1 [Reference]	NA	1 [Reference]	NA
≥ 1/2	5.36 (2.85–10.06)	< 0.001	1.35 (0.61–3.02)	0.4608
Histological type				
Endometrioid	1 [Reference]	NA	1 [Reference]	NA
Non-endometrioid	4.34 (2.19–8.58)	< 0.001	0.89 (0.28–2.89)	0.8523
Lymph node metastases				
No	1 [Reference]	NA	1 [Reference]	NA
Yes	4.83 (2.12–11)	< 0.001	2.96 (0.66–13.21)	0.155
LVSI				
Negative	1 [Reference]	NA	1 [Reference]	NA
Focal	6.58 (3.26–13.27)	< 0.001	3.03 (1.34–6.85)	0.0077
Substantial	35.8 (11.58–110.7)	< 0.001	0.24 (0.03–2.17)	0.2046
Primary tumor diameter, cm				
< 2	1 [Reference]	NA	1 [Reference]	NA
≥ 2	4.06 (1.44–11.43)	0.008	2.37 (0.71–7.87)	0.1604
Menopause				
No	1 [Reference]	NA	1 [Reference]	NA
Yes	2.08 (0.98–4.37)	0.055	NA	NA
Adenomyosis				
No	1 [Reference]	NA	1 [Reference]	NA
Yes	0.16 (0.02–1.18)	0.073	NA	NA

*AACCI* age-adjusted Charlson comorbidity index, *FIGO* federation of gynecology and obstetrics, *HR* hazard ratio, *LVSI* lymphatic vessel space invasion, *NA* not applicable

## Nomograms and model performance

**Fig. 2:**
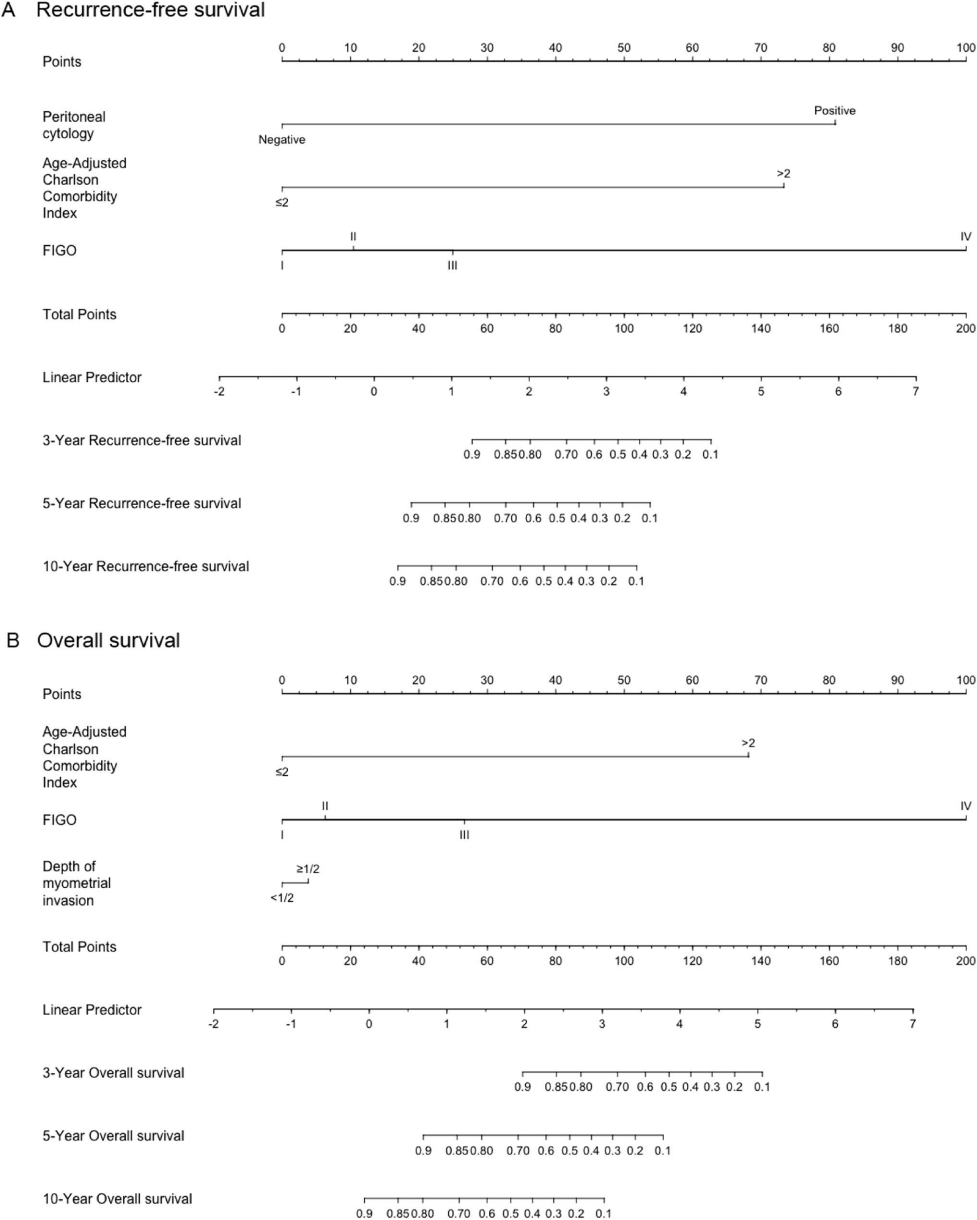
Nomograms predicting survival in patients after resection of endometrial cancer. Tips: A nomogram to predict recurrence-free survival was created based on three independent prognostic factors, and a nomogram to predict overall survival was created based on three prognostic factors (see the Model Specifications and Predictors of RFS (**A**) and OS (**B**) subsection of the Methods section).

**Fig. 3:**
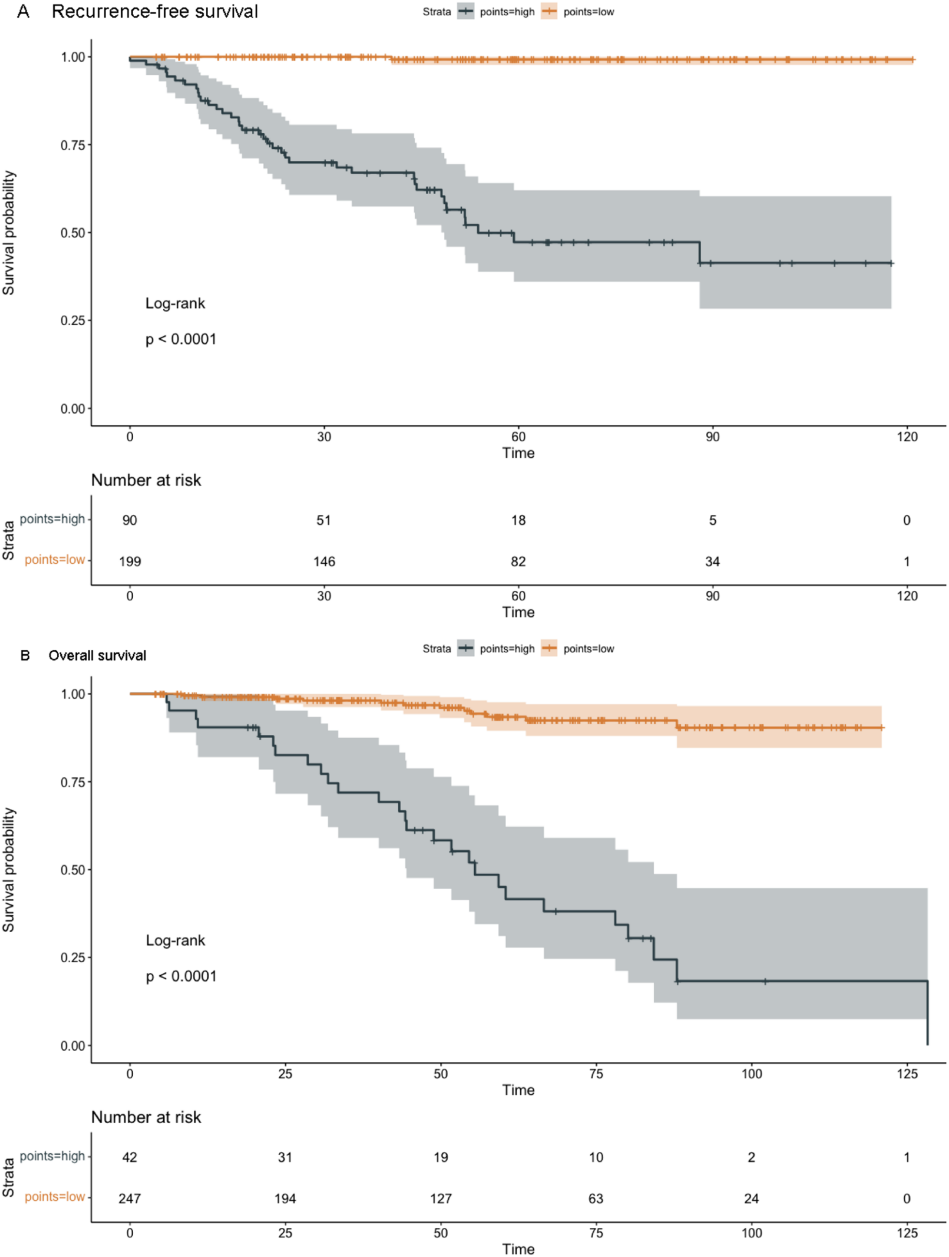
Kaplan–Meier curves demonstrating survival in patients after resection for endometrial cancer according to the cutoff point of predicted survival for RFS (**A**), the cutoff point is 179, and OS (**B**), the cutoff point is 140

**Fig. 4:**
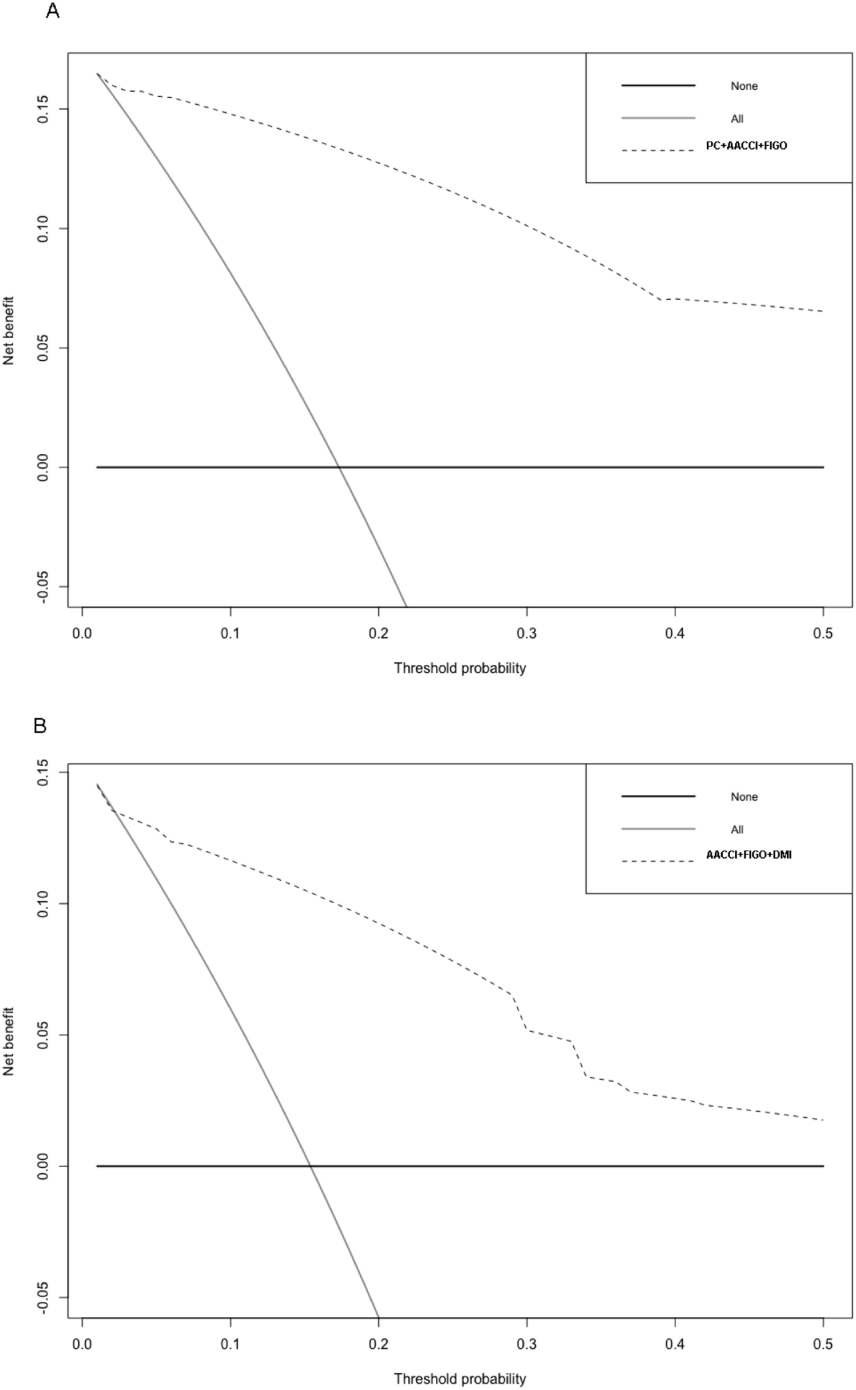
DCA analysis of the nomogram for **A** RFS and **B** OS. DCA: decision curve analysis; RFS: recurrence-free survival; OS: overall survival

The nomograms for predicting RFS and OS after surgical resection in patients with EC are shown in Fig. 2. The predictive nomogram for RFS was established using the following three independent predictive variables: AACCI (≤ 2 or  > 2), peritoneal cytology result (negative or positive), and FIGO stage (I–IV). The predictive nomogram for OS was established based on the following three prognostic factors: AACCI (≤ 2 or  > 2), FIGO stage (I, II, III, or IV), and depth of myometrial invasion (< 1/2 or  ≥ 1/2). Worse prognosis was correlated with greater total scores, which were derived by summing all the points for each element in the nomogram. For instance, a patient with a high AACCI (> 2), early FIGO stage (II), and negative peritoneal cytology result could score 83 points (73, 10, and 0 points for each item, respectively), yielding projected 3-, 5-, and 10-year RFS of 69.0%, 45.0%, and 38.0%, respectively. Similarly, a patient with a high AACCI (> 2), early FIGO stage (II), and depth of myometrial invasion < 1/2 could score 74 points (68, 6, and 0 points for each item, respectively), with 3-, 5-, and 10-year OS of 88.0%, 64.0%, and 38.0%, respectively. Calibration was further assessed by plotting Kaplan–Meier curves across the nomogram score cutoffs in the dataset (Fig. 3). Patients with higher scores indicating reduced projected 5-year RFS showed a significantly poorer prognosis (41.4%, 5-year RFS) than patients with lower points (99.2%, 5-year RFS) (*P* < 0.00001). Likewise, patients with high scores indicating lower projected 5-year OS showed a significantly poorer prognosis (41.6%, 5-year OS) than those with lower scores (92.4%, 5-year OS) (*P* < 0.00001). C-statistics was used to identify the discriminant ability of the RFS and OS models (0.895 for RFS and 0.891 for OS). Model accuracy and potential model overfitting were examined using bootstrap validation with 1000 re-samplings. A 39-sample bootstrap calibration plot was used to predict the 5-year RFS and OS (Online Resource 3). Given the inherent prognostic preference in our dataset, our calibration deviated significantly from the diagonal at lower predicted probability of actual occurrence. Therefore, our model may not be suitable for identifying patients with a low probability of actual occurrence. DCA, which is a simple technique used to evaluate the feasibility and clinical value of predictive technologies, showed that our constructed nomograms could better predict RFS and OS in patients with EC (Fig. 4).

**Fig. 5:**
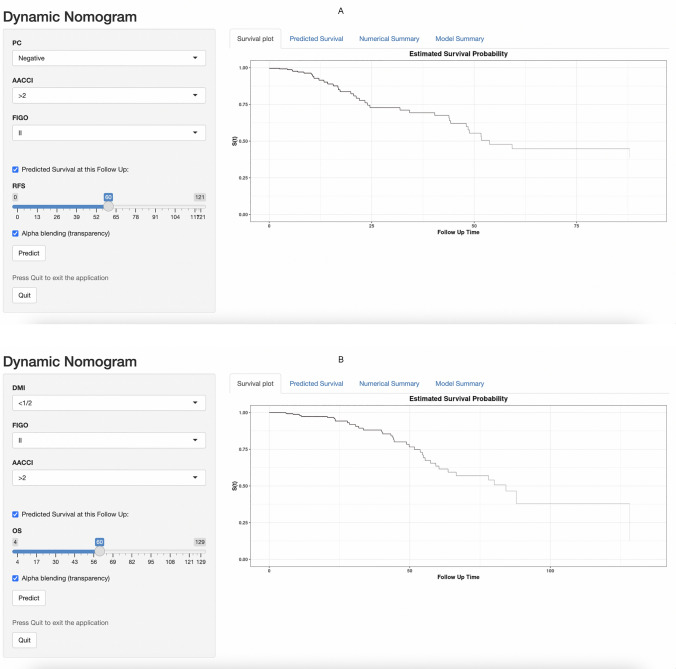
Examples of website usage. Entering the input value to predict **A** RFS and **B** OS. RFS: recurrence-free survival; OS: overall survival

Furthermore, we developed a website to enable clinicians to use the proposed models for individualized risk prediction. The online dynamic nomogram for RFS is accessible at https://dyyyfkhz.shinyapps.io/DynNomRFSapp/ and that for OS at https://dyyyfkhz.shinyapps.io/DynNomOSapp/ (Fig 5).

## Discussion

In this study, we proposed nomograms based on clinicopathological variables to predict RFS and OS in patients with EC after surgical resection. While total surgical resection remains the preferred course of action for EC, 65–85% of patients experience recurrence after surgery [18]. Recent advancements in detection and treatment technology have led to a decline in recurrence rates [19]. Approximately 70% of patients with EC have been reported to have stage I disease, with 5-year OS of 74–91%. However, the 5-year OS has been reported as 57–66% for stage III disease and only 20–26% for stage IV disease, suggesting that metastasis is associated with a poorer outcome [20, 21]. Prognostic heterogeneity following surgery for EC varies greatly depending on the tumor stage [22]. Accurate prognosis following surgery for EC is essential for providing patients with accurate information about their long-term prognosis and for selecting patients for adjuvant therapy. While the FIGO staging system is currently the most commonly used method, the reported prognostic factors vary, and the optimal approach to stratify the risk of EC patients remains unknown [23].

Herein, two nomograms were created to predict RFS and OS in individuals following resection for EC based on patient-related factors. This information aids in deciding personalized treatment and monitoring methods and provides insight into a patient's prognosis. Notably, the nomograms created herein were constructed using data spanning nearly a decade's worth of records of patients with EC who underwent surgery. Moreover, unlike the previously suggested nomograms based on the Surveillance, Epidemiology, and End Results program database [11], the effectiveness of the current nomograms has been thoroughly evaluated and internally validated. In addition, the present study on resection for EC included several previously mentioned variables that reportedly affected prognoses [24–26]. Various factors have been identified to be associated with outcomes, with little agreement on which factors determine prognosis [10, 26]. For example, some studies [10, 27, 28] have reported age, tumor stage, grade, LVSI, myometrial invasion, cervical stromal invasion, and tumor size. However, other researchers found no association between long-term survival and LVSI, histological type, menopause, and adenomyosis [29–32]. Similarly, we found no association between menopause, adenomyosis, and RFS or OS in the present study. Comparatively, several studies on EC have revealed that LVSI, advanced FIGO stage (III/IV), histological grade, myometrial invasion depth, cervical stromal invasion, and tumor size at diagnosis are associated with poorer outcomes [8, 27, 28, 33, 34]. Regarding mortality risk, patients with AACCI > 2 and primary tumor diameter > 2 cm had 93-fold and sixfold higher risks of death, respectively, and regarding recurrence risk, patients with AACCI > 2, stage IV disease and high histological grade had 52-fold, 138-fold, and threefold higher risks of recurrence, respectively. AACCI was identified as an independent risk variable for OS and RFS, consistent with previous conclusions [35].

The prognosis of tumors such as EC can be heterogeneous, underscoring the importance of accurate risk stratification of these patients. Nomograms may provide more individualized prognostic information to patients rather than the FIGO staging system, which relies on population-based data. Li et al. [11] proposed a nomogram for patients with EC that considered age, FIGO stage, histological grade, histological type, distant metastasis, and tumor stage, and we consider this model to lack convenience and practicality. Hence, we limited our model factors to focus solely on patients undergoing surgical treatment for EC. Nomograms stratified by cutoffs facilitated the identification of distinct patient groups with varying risks of relapse and mortality. Moreover, both nomograms showed good discriminatory capability, with C-statistics of 0.895 for predicting RFS and 0.891 for predicting OS. The nomograms also provided median 5-year survival predictions similar to the Kaplan–Meier curve survival estimates. These findings suggest that our proposed nomograms can offer patients with postoperative EC information on the risk of recurrence and survival. Compared with previous models [36], the IDI index evaluated the overall improvement of the model: 18.9% for 5-year and 17.3% for 10-year RFS prediction.

This study has some limitations. First, this was a single-institution retrospective study. Patient differences will affect the universality and prediction accuracy of the model. Second, the prognostic significance of OS may be compromised, as data on treatments, such as chemotherapy, radiotherapy, and surgery, which could influence patient survival, were excluded from the nomogram. Moreover, next-generation sequencing molecular classification has demonstrated superiority over the conventional pathological assessment of grade and histotype [37]. These factors should be considered for future modeling. Finally, although the proposed nomograms were internally validated using bootstrapping, additional studies are required for external validation.

In conclusion, this study is the first to develop online nomograms based on three variables to dynamically predict RFS and OS in women with EC. The proposed nomograms can be used to stratify patients into different predictive groups based on relapse and long-term outcomes. These novel models demonstrated superior performance and discriminative power, offering valuable information for gynecologists when designing customized clinical treatments. Clinicians can use our model through the web calculator we created. Additionally, the nomograms performed well when internally validated. External validation of the proposed nomograms is required to demonstrate their usefulness in assessing long-term prognosis following therapeutic EC excisions.

## Data Availability

The raw data supporting the conclusions of this article will be made available by Zhen Hu, without undue reservation.
